# Digital media use, depressive symptoms and support for violent radicalization among young Canadians: a latent profile analysis

**DOI:** 10.1186/s40359-024-01739-0

**Published:** 2024-05-10

**Authors:** Diana Miconi, Tara Santavicca, Rochelle L. Frounfelker, Aoudou Njingouo Mounchingam, Cécile Rousseau

**Affiliations:** 1 Department of Educational Psychology and Adult Education, University of Montréal, Montréal, QC Canada; 2 MUHC Research Institute, Montréal, QC Canada; 3 College of Health, Lehigh University, Bethlehem, PA USA; 4 Department of Sociology, Université de Québec à Montréal, Montréal, QC Canada; 5 Division of Social and Cultural Psychiatry, McGill University, Montréal, QC Canada

**Keywords:** Violent radicalization, Digital media use, Depression, Young people, Person-centered approach

## Abstract

**Background:**

Despite the prominent role that digital media play in the lives and mental health of young people as well as in violent radicalization (VR) processes, empirical research aimed to investigate the association between Internet use, depressive symptoms and support for VR among young people is scant. We adopt a person-centered approach to investigate patterns of digital media use and their association with depressive symptoms and support for VR.

**Methods:**

A sample of 2,324 Canadian young people (M_age_ = 30.10; SD_age_ = 5.44 ; 59% women) responded to an online questionnaire. We used latent profile analysis to identify patterns of digital media use and linear regression to estimate the associations between class membership, depressive symptoms and support for VR.

**Results:**

We identified four classes of individuals with regards to digital media use, named

Average Internet Use/Institutional trust, Average internet use/Undifferentiated Trust, Limited Internet Use/Low Trust and Online Relational and Political Engagement/Social Media Trust. Linear regression indicated that individuals in the Online Relational and Political Engagement/Social Media Trust and Average Internet Use/Institutional trust profiles reported the highest and lowest scores of both depression and support for VR, respectively.

**Conclusions:**

It is essential to tailor prevention and intervention efforts to mitigate risks of VR to the specific needs and experiences of different groups in society, within a socio-ecological perspective. Prevention should consider both strengths and risks of digital media use and simulteaneously target both online and offline experiences and networks, with a focus on the sociopolitical and relational/emotional components of Internet use.

## Background

The recent increase in support for – and direct engagement in – ideologically motivated violence among youth can be associated with the increase in social polarization in society [1] as well as the specificities of adolescence and early adulthood, a seminal period for the development of ideologies [2, 3]. Violent radicalization (VR) is a complex and multidimensional phenomenon [4] defined as a process whereby an individual or a group increases support for violence as a legitimate means to reach a specific (e.g., political, social, religious) goal [5]. Noteably, VR processes are increasingly occurring online [6, 7]. Internet use has been primarily investigated in the field of terrorism studies and with samples of radicalized individuals [6, 8]. Less is known about the association of digital media use, social polarization and attitudes towards support for VR among young people. Although the association between attitudes and behaviors is not a linear one, positive attitudes towards VR can contribute to the creation of socially polarized environments that fuel conflicts and shatter social solidarities, resulting in some cases in extremist ideologies and the normalization of violence. In such contexts, vulnerable individuals - such as those experiencing significant social grievances - are at higher risk of engaging in violent acts and extremism. Thus, in a primary prevention perspective, a reduction in support of VR among youth can result in an overall decrease of violence in our societies in the short and long-term [9–11].

Although numerous interventions target online literacy and social media use as potential ways to counter violent extremism [7], empirical research on their effectiveness is scarce and the role that Internet use plays in the development of positive attitudes towards VR among young people is largely understudied. While depressive symptomatology, which has also been increasing among young people in the past decade [12, 13], is associated with both digital media use and support for VR [14–17], empirical research has not yet examined the associations between these variables simultaneously in one study. The current study aims to fill this gap in the literature by empirically investigating if and how patterns of digital media use are differently associated with depressive symptoms and support for VR among a sample of Canadian young people via a person-centered approach. Given the prominent role that digital media use play in both VR processes and mental health among young people, a better understanding of risk and protective factors associated with digital media use is warranted to inform and tailor evidence-based prevention programs that could significantly help reduce social ruptures and the associated risk of violence.

### Digital media use and support for VR

The online space has become a central developmental context for young people [18, 19]. Empirical evidence remains mixed, suggesting that digital media use can be either a risk or protective factor across multiple developmental outcomes depending on a complex interplay between both online and offline factors [18]. A consensus is now emerging that the specific behaviors in which youth engage online, rather than overall digital media per se, are key determinants of well-being. Yet, gaps in knowledge remain [20].

On the one hand, digital media can be used to connect with peers and to counter isolation, thus extending or reinforcing one’s social support network and possibly one’s trust in institutions and in democracy. On the other hand, the Internet can provide instant and unfiltered access to content and groups that propagate fake news, extreme beliefs and encourage violent actions, representing one of the main settings that can facilitate disaffiliation phenomena and recruitment of young people by extremist groups [7, 21–24]. Notably, whereas the majority of young people go online, only a minority of them get involved in VR processes. As such, it is likely that digital media use does not have a linear relationship with support for VR, but that specific constellations of digital media use are differentially associated with support for VR [8, 25].

Young people’s use of digital media is complex and heterogeneous [18], making the measurement and conceptualization of digital media use a challenging area [26, 27]. In this study, we focus on some aspects of digital media use that have been theoretically and/or empirically associated with VR, namely time on social media, reasons for Internet use (work, informational, entertainment, social), news literacy, trust in specific online sources of information (news, peers, influencers, government, youtube), preference for online social interactions and online political interactions.

The Internet can be used for multiple purposes, spanning from work or entertainment, to relational maintenance and social interaction [18, 28]. Although spending more time online has been associated with increased exposure to extremist content [23], whether this exposure is associated with risks of VR is yet unclear [29]. Overall, the impact of time spent on social media on a variety of social and health outcomes including VR varies based on the specific online activities and experiences [8, 18, 20].

Of importance, the Internet is currently the most important source of information for young people [30, 31], but trust on the validity of information from official governmental websites as well as from social media (e.g., Instagram, Twitter, Youtube) can vary between individuals. Misinformation and beliefs in conspiracy theories have been associated with higher support for VR [32, 33]. News literacy is considered a potential avenue to countering both misinformation, social polarization and online extremism [34, 35]. News literacy is defined as the ability to find/identify/recognize news, critically evaluate and produce them [36]. However, empirical research that examines the association between news literacy and support for VR is lacking.

Prior research has found that preference for online social interactions over face-to-face relationships represents a risk factor for support for VR [37, 38]. Preference for online social interactions is characterized by beliefs that one is safer, more confident, more comfortable and appreciated when online as opposed to offline [39] and is considered a component of problematic Internet use as it implies problematic relational experiences offline.

Some studies suggest that actively seeking and engaging with extremist content online is associated with higher risk of VR [8, 22, 25]. Although online interactions with strangers have been associated with higher risk of psychological distress [17, 40], the extent to which interactions with known and unknown people around political or current issues are associated, if at all, with support for VR has yet to be explored [23].

Given the variety in online experiences and type of digital media use, a person-centered approach via a latent profile analysis (LPA) facilitates examining different constellations of digital media use among young adults and associations between latent groups and support for VR. As VR is the result of complex and unique interplays between personal and social/contextual variables [4, 41, 42], identifying patterns of vulnerabilities online via a person-centered approach can inform the development of tailored VR prevention programs targeting digital media use.

### The present study

The present study adopts a person-centered approach to investigate: 1) patterns of digital media use among young Canadians. Specifically we focus on reasons for digital media use (i.e., work, entertainment, socialization, information), reported trust in different sources of online information (i.e., official government and news websites and social media), news literacy, time on social media, preference for online social interactions and online political interactions (e.g., posting/discussing with peers vs strangers, having conflicts online about these issues); 2) the association between patterns of digital media use and levels of depressive symptoms; and 3) the association between patterns of digital media use and support for VR. We expect to identify at least two groups of young people who differ in their reported digital media use. Given that we do not have a priori knowledge of the class structure in the data, we did not have a priori hypotheses about the association between each profile and depressive symptoms. However, we anticipate that the group(s) that will report the highest levels of depressive symptoms will also be at higher risk of supporting VR.

## Method

### Participants

A total of 2,695 participants answered an online survey; missing outcome data (*n*=362) and individuals identifying as “other” gender (*n*=9) were removed for methodological concerns given the very small sample size of this gender group. Final sample size was 2,324 participants (59.3% women; mean age = 30.10; SD = 5.44 ). Socio-demographic characteristics are presented in Table 1.

**Table 1:** Characteristics of Study Participants (*N* = 2,324)

**Variable**	**Total (*N*=2324)**
**Gender**	
Woman	1379 (59.3%)
Man	942 (40.5%)
Missing	3 (0.1%)
**Education**	
None/Less than high school	35 (1.5%)
High school graduate	322 (13.9%)
Apprenticeship, technical institute, trade or vocational school (any year)	138 (5.9%)
College, CEGEP or other non-university certificate or diploma (any year)	496 (21.3%)
University certificate, diploma or degree (any year)	1328 (57.1%)
Missing	5 (0.2%)
**Income**	
$19,999 or less	115 (4.9%)
Between $20,000 and $39,999	278 (12.0%)
Between $40,000 and $59,999	326 (14.0%)
Between $60,000 and $79,999	356 (15.3%)
Between $80,000 and $99,999	373 (16.1%)
$100,000 or more	683 (29.4%)
Missing	193 (8.3%)
**Employment**	
Not employed	429 (18.5%)
Employed - essential	973 (41.9%)
Employed - non essential	894 (38.5%)
Missing	28 (1.2%)
**Generation**	
Third or more	1361 (58.6%)
First	427 (18.4%)
Second	529 (22.8%)
Missing	7 (0.3%)
**Province**	
Alberta	521 (22.4%)
Ontario	936 (40.3%)
Quebec	867 (37.3%)
**Religion**	
No religion	1171 (50.4%)
Religion	1078 (46.4%)
Missing	75 (3.2%)
**Age**	
Mean (SD)	30.1 (5.43)
Median [Min, Max]	30.0 [18.0, 41.0]
**Depression**	
Below clinical cut-off	1191 (51.2%)
Above Clinical cut-off	1048 (45.1%)
Missing	85 (3.7%)
**Time Spent On Social Media (daily)**	
Less than 2 hours	894 (38.5%)
2- 4 hours	886 (38.1%)
4-6 hours	366 (15.7%)
6 or more hours	176 (7.6%)
Missing	2 (0.1%)
**Reasons for Internet Use**	
**Personal Relationships**	
Mean (SD)	3.06 (1.07)
Median [Min, Max]	3.00 [1.00, 5.00]
Missing	3 (0.1%)
**Actively search for information/news**	
Mean (SD)	3.07 (1.06)
Median [Min, Max]	3.00 [1.00, 5.00]
Missing	3 (0.1%)
**Entertainment**	
Mean (SD)	3.76 (0.965)
Median [Min, Max]	4.00 [1.00, 5.00]
Missing	1 (0.0%)
**Work**	
Mean (SD)	2.80 (1.38)
Median [Min, Max]	3.00 [1.00, 5.00]
Missing	11 (0.5%)
**Online interactions around politics/current affairs**	
**Posted information on social media**	
Mean (SD)	1.70 (1.16)
Median [Min, Max]	1.00 [1.00, 6.00]
Missing	6 (0.3%)
**Discussed with people you know**	
Mean (SD)	2.67 (1.31)
Median [Min, Max]	2.00 [1.00, 6.00]
Missing	7 (0.3%)
**Discussed with people you do not know**	
Mean (SD)	1.70 (1.16)
Median [Min, Max]	1.00 [1.00, 6.00]
Missing	11 (0.5%)
**Had verbal conflicts with known people around information shared/posted online**	
Mean (SD)	1.49 (0.993)
Median [Min, Max]	1.00 [1.00, 6.00]
Missing	15 (0.6%)
**News Literacy**	
Mean (SD)	21.4 (4.43)
Median [Min, Max]	22.0 [6.00, 30.0]
Missing	145 (6.2%)
**Trust on sources of information online**	
**News**	
Mean (SD)	3.14 (0.760)
Median [Min, Max]	3.00 [1.00, 4.00]
Missing	13 (0.6%)
**Peers**	
Mean (SD)	2.50 (0.815)
Median [Min, Max]	3.00 [1.00, 4.00]
Missing	13 (0.6%)
**Influencers**	
Mean (SD)	2.06 (0.842)
Median [Min, Max]	2.00 [1.00, 4.00]
Missing	13 (0.6%)
**Government**	
Mean (SD)	3.31 (0.794)
Median [Min, Max]	3.00 [1.00, 4.00]
Missing	13 (0.6%)
**Youtube**	
Mean (SD)	2.50 (0.816)
Median [Min, Max]	3.00 [1.00, 4.00]
Missing	11 (0.5%)
**P**r**eference for Online Social Interactions**	
Mean (SD)	42.4 (16.1)
Median [Min, Max]	42.0 [0, 91.0]
Missing	34 (1.5%)
**Radicalism Intention Scale (RIS)**	
Mean (SD)	10.8 (6.12)
Median [Min, Max]	9.00 [4.00, 28.0]

### Procedure

Data were collected in November 2021, during the COVID-19 pandemic in Alberta, Ontario, and Quebec. Participants were recruited through the Leger360 online platform with over 500,0000 registered members and answered the survey in either English or French [43]. informed consent to participate was obtained electronically from all of the participants in the study, and response rate was 53.8%. Exclusion criteria were individuals under the age of 18 or above 41. Study protocol and procedures were approved by the Institutional Review Board of.

## Measures

### Support for VR

The Radicalism Intention Scale (RIS) is a 4-item subscale of the Activism and Radicalism Intention Scales (ARIS) [44]. It assesses an individual’s readiness to participate in illegal and violent behavior in the name of one’s group or organization. Respondents rated their agreement with four statements on a seven-point Likert scale, with higher scores indicating more support for VR (range 4-28). The scale has good psychometric properties among young adults [45] (α = .89; Ω = 0.89).

### Time spent on social media (daily)

Participants were asked to identify how many hours they spend on social media on a typical day (i.e., less than 2 hours, 2-4 hours, 4-6 hours, and 6 hours or more).

### Reasons for Internet use

Four statements on Internet use were presented (i.e., using Internet: for personal relationships, to actively search for information/news, for entertainment, and for work). Participants were asked to indicate on a 5-point Likert scale how much they used the Internet for each reason (not at all, a little, moderately, a lot, most of the time).

### News literacy

Was measured as a subscale of the literacy scale by Jones-Jang et al. [36]. Participants were asked using a 5-point Likert scale how much they agreed with each statement (six items, from 1-strongly disagree to 5- strongly agree, range 6 - 30)(α = .80; Ω = 0.80).

### Trust on online sources of information

Five statements around trusting different sources of online information were presented, namely trust in news, peers, influencers, government, and YouTube sources of information. Participants were asked to indicate how often they trust each source of information on a 4-point Likert scale (never, rarely, sometimes, often).

### Preference for online social interactions

(PFOSI) was measured with the 13-item Social Comfort subscale of the Online Cognition Scale [46]. Participants rated on a 8-point Likert scale (range 0 – 91) how much they agreed with statements describing their relationships with people who they know primarily through the Internet (e.g., chat rooms, message boards, online gaming communities). Higher scores indicate more preference for online social interactions (α = .92; Ω = 0.92).

### Online political interactions

Participants were asked to indicate on a 6-point Likert scale (from “None/No time at all”, to “Several times a day”) how often their online interactions were oriented around these four statements: posted information about politics/current affairs on social media, discussed politics/current affairs with people you know, discussed politics/current affairs with people you do not know, had verbal conflicts with known people around information shared/posted online.

### Depressive symptoms

Depressive symptoms were measured by using the 15-item subscale of the Hopkins Symptom Checklist-25 (HSCL-25) [47]. Items are rated on a Likert scale from 1 (not at all) to 4 (extremely) based on how much discomfort that problem has caused them during the past seven days, and a total score is obtained by computing the mean of all items. The clinical cut-off is set at 1.75 (score range from 1 to 4) and scores have been recoded as below (0) or above (1) this cut-off. The HSCL-25’s psychometric qualities have been well established [48] (α = .94; Ω = 0.94).

### Socio-demographic variables

Participants provided information on their age, gender (man or woman), education (None/Less than high school, High school graduate, Apprenticeship, technical institute, trade or vocational school, College, CEGEP or other non-university certificate or diploma or University certificate, diploma or degree), Income ($19,999 or less, $20,000- $39,999, $40,000- $59,999, $60,000 - $79,999, $80,000- $99,999, $100,000 or more), employment (not employed, employed -essential, employed – non-essential), generational status (first-generation immigrant, second-generation immigrant, and third generation or more immigrant/non-immigrant), province (Alberta, Ontario, Quebec), religious beliefs (no religion, religion), and age.

## Statistical analyses

Analyses were conducted using *R* software [49]. Missing data were imputed using the Random Forest method via the *mice* package [50, 51]. Sensitivity analysis suggested that missing data and multiple imputations did not alter the observed patterns of associations. First, we estimated the LPA model around variables related to digital media use via the *tidyLPA* package [52]. LPA is an analytic strategy that attempts to identify subgroups of people within a heterogeneous population who has a high degree of homogeneity in responses on a set of indicators. The appropriate number of latent profiles was selected based on the Akaike information criterion (AIC), the Bayesian information criterion (BIC), the Sample-size-adjusted BIC (SABIC), Bootstrap Likelihood Ratio Test (BLRT), characteristics of the profiles (interpretability of response profiles or uniqueness) and a conservative profile sample size (>10%) [53–56]. Lower AIC, BIC and SABIC values and a statistically significant BLRT indicate a better model fit [53, 54]. Once the best LPA solution was identified, the level of entropy (acceptable if >.70) and Average Posterior Class Probability (AvePP; acceptable if >.70) were examined to determine the accuracy of classification [57].

Next, based on the predicted probabilities of profile membership made by the LPA, we assigned each participant to a specific profile. Analyses were then conducted on the univariable associations between sociodemographic characteristics and profile membership. Frequencies of profile membership by sociodemographic characteristics can be found in Table 3.

Lastly, we conducted linear regression analyses that estimated support for VR as a function of profile membership. A sequential model building approach was used to evaluate the associations between profiles and support for VR. Model 1 presents the unadjusted association between profile and support for VR; model 2 adjusts for sociodemographic characteristics, and model 3 adjusts for sociodemographic characteristics and depression.

## Results

### Latent Profile Structure

**Fig. 1:**
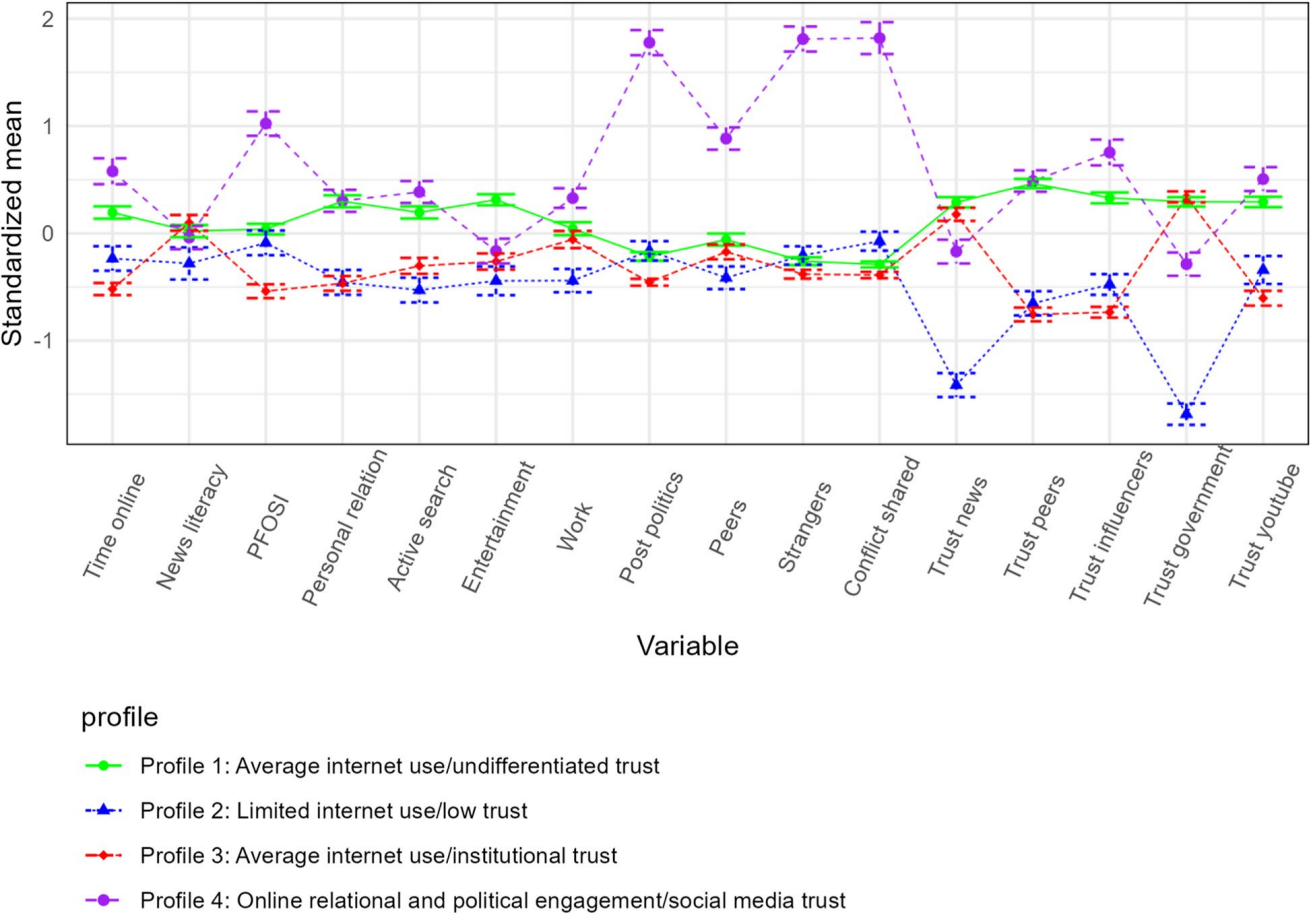
Four-Profile Solution with Standardized Mean by Item Responses (*N* = 2324) Note. *PFOSI* Preference for online social interactions

LPA models 2 through 6 are presented in Table 2 along associated BIC and log-likelihood values. The four-class solution was selected as the best fit for sample size of profiles (>10%) and interpretability of findings, despite not having the lowest BIC value. Figure 1 presents profile membership item response probabilities for digital media use. Participants in all profiles had a high probability of reporting average levels of news literacy. Unique class characteristics emerged around time spent on social media and overall Internet use preference for online social interactions, online political interactions and trusting multiple sources of information. Profile 1, named *Average Internet use/Undifferentiated trust* is characterized by individuals who demonstrated average Internet use yet infrequently used the Internet for interactions around politics/current affairs and showed undifferentiated trust towards information found on-line, regardless of the source. Participants in Profile 2, named *Limited Internet use/Low Trust*, infrequently used the Internet across the considered reasons and reported a low probability of trusting news and government sources compared to information from social media (e.g., peers, influencers, youtube). Profile 3, named *Average Internet use/Institutional trust*, is characterized by participants with average and undifferentiated internet use, who were more likely to report greater trust in institutional sources of online information (e.g., news, government) compared to other social media sources. Profile 4, named *Online relational and political engagement/Social media trust*, consists of individuals with a high probability of preferring online, as opposed to in person, social interactions and spending a large amount of time on social media on a daily basis. In addition, participants in Profile 4 had a high probability of using the Internet for discussing politics and other issues with both peers and strangers, actively posting on-line about politics, and were more likely to report conflicts online compared to all other profiles. Profile 4 participants had a lower probability of trusting news and government sources compared to other sources of information online (peers, influencers, youtube). Overall, Profile 1 and 3 included 46.8% and 27.9% of participants, respectively. Profile 2 was smaller and included 11.4% of participants, while Profile 4 included 13.9% of participants.

**Table 2:** Fit statistics for solutions with 2 to 6 profiles on multiple imputed dataset (*N* = 2324)

Profile solutions	Profile sizes	AIC	BIC	SABIC	BLRT	BLRT *p*-value
2	1, *n*=1964(84.64%)	100712.0	101518.7	100838.1	4893.86	0.01
	2, *n*=357(15.36%)					
3	1, *n*=1353 (58.22%)	99476.45	99856.02	99646.32	1269.25	0.01
	2, *n*=343(14.76%)					
	3, *n*=628 (27.02%)					
**4**	**1, *n*=1088 (46.82%)**	**98894.71**	**99372.04**	**99108.34**	**629.85**	**0.01**
	**2, *n*= 266 (11.45%)**					
	**3, *n*=648 (27.88%)**					
	**4, *n*=322(13.86%)**					
5	1, *n*=715(30.77%)	98410.99	98986.1	98668.38	839.76	0.01
	2, *n*=552(23.75%)					
	3, *n*=317(13.64%)					
	4, *n*=530(22.81%)					
	5, *n*=210(9.04%)					
6	1, *n*=194 (8.35%)	97579.65	98252.52	97880.79	578.15	0.01
	2, *n*=557(23.97%)					
	3, *n*=231(9.94%)					
	4, *n*=565(24.31%)					
	5, *n*=184(7.92%)					
	6, *n*=593(25.52%)					

*AIC* Akaike Information Criterion, *BIC* Bayesian information Criterion, *SABIC* Sample-size-adjusted BIC, *BLRT* Bootstrapped likelihood ratio test. The Model with 4 profiles (bold) has been selected as the best profile solution where all profiles include at least 10% of sample

### Profile belonging, sociodemographic characteristics and depressive symptoms

Table 3 represents sociodemographic characteristics and depressive symptoms by profile for study participants. All variables were significantly associated (*p* < 0.05) with profile belonging at the univariable level. The *Average Internet use/Undifferentiated trust* profile included a higher representation of women, non-immigrant, employed and non-religious participants, as well as participants who reported high education and income. A total of 45% of participants in this profile scored above the clinical cut-off for depressive symptoms. Participants in the *Limited Internet use/Low Trust* profile had a higher probability of being less educated, reporting a lower income and more unemployement. Participants in this profile were more likely to live in Alberta. Profile 3, *Average Internet use/Institutional trust* included participants who were highly educated, had high income, without an immigration background (third generation or more) and without a religion. Participants in this profile reported also the lowest levels of depression (37.5% above clinical cut-off) and more of them lived in Quebec. Finally, the *Online relational and political engagement/Social media trust* profile had an overrepresentation of men, immigrants, participants with a religion and who lived mainly in Ontario. In addition, participants in this group were overall educated but reported low income and high unemployment. A total of 70% of participants in this profile scored above the clinical cut-off to our measure of depressive symptoms.

**Table 3:** Frequencies of Profile Membership by Sociodemographic Characteristics and Depressive Symptoms (*N* = 2,324)

	**Profile 1:** **Average internet use/Undifferentiated Trust** **(*N*=1088)**	**Profile 2:** **Limited Internet Use/Low Trust** **(*N*=266)**	**Profile 3:** **Average Internet Use/Institutional trust** **(*N*=648)**	**Profile 4:** **Online Relational and Political Engagement/Social Media Trust** **(*N*=322)**	**Total (*N*=2324)**	χ^**2**^** (*df*)**
**Gender**						102.47(3)***
Woman	746 (68.6%)	152 (57.1%)	360 (55.6%)	123 (38.2%)	1381 (59.4%)	
Man	342 (31.4%)	114 (42.9%)	288 (44.4%)	199 (61.8%)	943 (40.6%)	
**Education**						55.89(12)***
None/Less than high school	16 (1.5%)	7 (2.6%)	5 (0.8%)	7 (2.2%)	35 (1.5%)	
High school graduate	149 (13.7%)	59 (22.2%)	71 (11.0%)	44 (13.7%)	323 (13.9%)	
Apprenticeship, technical institute, trade or vocational school (any year)	58 (5.3%)	28 (10.5%)	33 (5.1%)	20 (6.2%)	139 (6.0%)	
College, CEGEP or other non-university certificate or diploma (any year)	222 (20.4%)	69 (25.9%)	137 (21.1%)	69 (21.4%)	497 (21.4%)	
University certificate, diploma or degree (any year)	643 (59.1%)	103 (38.7%)	402 (62.0%)	182 (56.5%)	1330 (57.2%)	
**Income**						57.41(15)***
$19,999 or less	60 (5.5%)	22 (8.3%)	20 (3.1%)	21 (6.5%)	123 (5.3%)	
Between $20,000 and $39,999	150 (13.8%)	42 (15.8%)	57 (8.8%)	54 (16.8%)	303 (13.0%)	
Between $40,000 and $59,999	155 (14.2%)	51 (19.2%)	89 (13.7%)	53 (16.5%)	348 (15.0%)	
Between $60,000 and $79,999	163 (15.0%)	49 (18.4%)	119 (18.4%)	57 (17.7%)	388 (16.7%)	
Between $80,000 and $99,999	185 (17.0%)	31 (11.7%)	131 (20.2%)	62 (19.3%)	409 (17.6%)	
$100,000 or more	375 (34.5%)	71 (26.7%)	232 (35.8%)	75 (23.3%)	753 (32.4%)	
**Employment**						16.39(6)*
Not employed	199 (18.3%)	63 (23.7%)	103 (15.9%)	70 (21.7%)	435 (18.7%)	
Employed - essential	446 (41.0%)	122 (45.9%)	288 (44.4%)	127 (39.4%)	983 (42.3%)	
Employed - non essential	443 (40.7%)	81 (30.5%)	257 (39.7%)	125 (38.8%)	906 (39.0%)	
**Generation**						47.55(6)***
Third or more	641 (58.9%)	172 (64.7%)	412 (63.6%)	139 (43.2%)	1364 (58.7%)	
First	203 (18.7%)	38 (14.3%)	96 (14.8%)	92 (28.6%)	429 (18.5%)	
Second	244 (22.4%)	56 (21.1%)	140 (21.6%)	91 (28.3%)	531 (22.8%)	
**Province**						25.89(6)***
Alberta	237 (21.8%)	77 (28.9%)	148 (22.8%)	59 (18.3%)	521 (22.4%)	
Ontario	447 (41.1%)	99 (37.2%)	230 (35.5%)	160 (49.7%)	936 (40.3%)	
Quebec	404 (37.1%)	90 (33.8%)	270 (41.7%)	103 (32.0%)	867 (37.3%)	
**Religion**						36.04(3)***
No religion	586 (53.9%)	142 (53.4%)	358 (55.2%)	117 (36.3%)	1203 (51.8%)	
Religion	502 (46.1%)	124 (46.6%)	290 (44.8%)	205 (63.7%)	1121 (48.2%)	
**Depression**						95.86(3)***
Below clinical cut-off	598 (55.0%)	132 (49.6%)	405 (62.5%)	96 (29.8%)	1231 (53.0%)	
Above clinical cut-off	490 (45.0%)	134 (50.4%)	243 (37.5%)	226 (70.2%)	1093 (47.0%)	
						***F-value (df)***
**Age**						19.22(3)***
Mean (SD)	29.4 (5.40)	30.6 (5.13)	31.3 (5.33)	29.8 (5.54)	30.1 (5.43)	
Median [Min, Max]	30.0 [18.0, 41.0]	31.0 [18.0, 41.0]	31.5 [18.0, 41.0]	30.0 [18.0, 41.0]	30.0 [18.0, 41.0]	

* *p* ≤ 0.05

*** *p* ≤ 0.001

### Associations of profile membership with support for VR

Profile membership was associated with scores on the RIS (*p* < 0.001). Participants in the *Online relational and political engagement/Social media trust* profile were more likely to report higher levels of support for VR compared to the other profiles in both unadjusted and adjusted models. Specifically, belonging to this profile was associated with a 0.91 (*SE* = 0.06, *p* < 0.001) increase in support for VR compared to the *Average Internet use/Undifferentiated trust* profile when controlling for sociodemographic variables and depressive symptoms (Table 4). Belonging to the *Average Internet use/Institutional trust* profile was associated with a -0.267 (*SE* = 0.046, *p* < 0.001) decrease in support for VR compared to the *Average Internet use/Undifferentiated trust* profile when controlling for sociodemographic variables and depression (Table 4). Gender, generation, province, age, and depressive symptoms were also associated with support for VR (*p* < 0.05). Men, first generation immigrants, participants from Ontario, younger participants, and participants reporting more depressive symptoms were more likely to report higher support for VR. Religion, income and education were not significantly associated with support for VR (Table 4).

**Table 4:** Associations between profile membership and support for Violent Radicalization as dependent variable

	**Model 1**					**Model 2**					**Model 3**				
**Variable**	**β**	**SE**	***p***	***F value (df)***	**η**^**2**^	**β**	**SE**	***p***	***F value (df)***	**η**^**2**^	**β**	**SE**	***p***	***F value (df)***	**η**^**2**^
**Profile Membership**				174.22(3)***	0.184				138.916(3)***	0.140				121.403(3)***	0.120
Average internet use/ Undifferentiated Trust	1					1					1				
Limited Internet Use/Low Trust	0.275	0.027	< 0.001			0.250	0.062	< 0.001			0.242	0.061	< 0.001		
Average Internet Use/Institutional trust	-0.300	0.062	< 0.001			-0.277	0.045	< 0.001			-0.267	0.046	< 0.001		
Online Relational and Political Engagement/Social Media Trust	1.072	0.045	< 0.001			0.964	0.058	< 0.001			0.906	0.059	< 0.001		
**Gender**									39.253(1)***	0.013				44.912(1)***	0.015
Woman						1					1				
Man						0.253	0.040	< 0.001			0.270	0.040	< 0.001		
**Education**									1.298(4)	0.002				1.006(4)	0.001
None/Less than high school						1					1				
High school graduate						0.180	0.159	0.256			0.146	0.158	0.354		
Apprenticeship, technical institute, trade or vocational school (any year)						0.246	0.170	0.146			0.194	0.168	0.250		
College, CEGEP or other non-university certificate or diploma (any year)						0.217	0.157	0.166			0.181	0.156	0.245		
University certificate, diploma or degree (any year)						0.136	0.155	0.378			0.105	0.154	0.494		
**Income**									1.973(5)	0.003				1.270(5)	0.002
$19,999 or less						1					1				
Between $20,000 and $39,999						-0.086	0.098	0.367			-0.091	0.095	0.340		
Between $40,000 and $59,999						-0.029	0.094	0.757			-0.017	0.094	0.854		
Between $60,000 and $79,999						-0.135	0.094	0.151			-0.121	0.094	0.198		
Between $80,000 and $99,999						-0.145	0.094	0.125			-0.116	0.094	0.215		
$100,000 or more						-0.188	0.091	0.038			-0.146	0.090	0.106		
**Employment**									3.631(2)*	0.002				3.050(2)*	0.002
Not employed						1					1				
Employed - essential						0.077	0.050	0.192			-0.050	0.054	0.353		
Employed – non-essential						-0.150	0.053	0.464			0.052	0.055	0.343		
**Generation**									3.720(2)*	0.003				4.570(2)*	0.003
Third or more						1					1				
First						0.135	0.053	0.011			0.147	0.052	0.005		
Second						0.086	0.049	0.079			0.097	0.048	0.046		
**Province**									12.285(2)***	0.008				9.254(2)***	0.006
Alberta						1					1				
Ontario						0.080	0.051	0.124			0.079	0.050	0.111		
Quebec						-0.143	0.055	0.005			-0.118	0.053	0.026		
**Religion**									1.248(1)	0.000				1.170(1)	0.000
No religion						1					1				
Religion						-0.042	0.040	0.264			-0.040	0.037	0.280		
**Age**						-0.015	0.039	< 0.001	17.860(1)***	0.006	-0.014	0.004	< 0.001	15.690(1)***	0.005
**Depression**														33.689(1)***	0.011
Below Clinical cut-off											1				
Above Clinical cut-off											0.224	0.039	< 0.001		

p-value * < 0.05 ** < 0.01 *** < 0.001

## Discussion

The current study investigated patterns of digital media use in a sample of young adults from three Canadian provinces. In addition, we examined whether these patterns were differentially associated with depressive symptoms and support for VR. Four profiles emerged from our LPA, confirming the pertinence of using a person-centered approach to shed light on the complex patterns of digital media use among young people. Overall, profiles differentiated participants mostly in terms of trust on specific sources of information and level and type of online engagement.

The two largest profiles (*Average Internet use/Undifferentiated trust* and *Average Internet use/Insitutional trust)* differed primarily in their trust of online sources of information. Specifically, individuals in the *Average Internet use/Insitutional trust* profile reported to trust more frequently institutional sources of information (i.e., government and news) rather than social media (i.e., youtube, influencers, and peers), suggesting an overall acceptance of mainstream information and of the status quo. In contrast, the *Average Internet use/Undifferentiated trust* group showed average levels of trust to all sources of information alike. This group spent slightly more time online than the *Average Internet use/Insitutional trust* one*,* but overall these two groups did not differ much in their online social or political interactions. These two groups included 74.7% of participants, indicating a divide in the population mostly linked to what online sources to trust for information. The remaining participants were equally distributed between the *Online relational and political engagement/Social media trust* and the *Low Internet use/Low trust* profiles. Participants in both of these profiles trusted more frequently alternative social media sources of information compared to institutional ones, but they differed in overall levels of trust, with the *Limited Internet use/Low trust* group reporting overall low levels of trust, especially for institutional sources of information. Participants in the *Online relational and political engagement/Social media trust* profile reported high levels of trust in alternative social media sources of information and were more actively and politically engaged online with both peers and especially with strangers. They spent more time online and preferred online social interactions more compared to the other profiles. Taken together, these findings suggest that patterns of digital media use echo the increasing polarization in our societies [58, 59] around issues of trust/distrust, engagement/disengagement as well as a variety of negative/positive online experiences. Indeed, the most important variables to differentiate the four profiles were related to the frequency of trusting different online sources of information as well as specific social and political interactions online, rather than reasons for Internet use or news literacy, which on the contrary did not seem to play a significant role in determining profile membership.

We suggest that the divide around trust in online information and engagement needs to be situated in the broader socio-political context, which can partly explain the socio-demographic differences we found across profiles.The *Average Internet use/Insitutional trust* and the *Average Internet use/Undifferentiated trust* profiles consisted of more affluent and more educated participants, mostly employed and without an immigration background. Participants in these profiles may benefit from more privileges in society, which can favor their trust in mainstream institutional sources of information online [60–62] . Indeed, participants in the *Average Internet use/Institutional trust* group were more likely to report the highest levels of education and income as well as the lowest levels of depressive symptoms followed by the *Average Internet use/Undifferentiated trust* profile. The difference in levels of depression between these two profiles can also be associated with the presence of younger participants and more women in the *Average Internet use/Undifferentiated trust* profile compared to the *Average internet use/Institutional trust* one. The *Low Internet use/Low trust* and *Online relational and political engagement/Social media trust* profiles included more participants reporting lower income. Participants in the *Online relational and political engagement/Social media trust* group included a higher percentage of men, participants with an immigration background and professing a religion – although participants in this profile reported an education level similar to the two larger profiles. This profile reported concerning levels of depression (70.2% above clinical cut-off). Relying on the internet for relational and political purposes combined with more frequent trust in alternative social media sources of information and less privileges in society can jeopardize young people’s mental health. Within a socio-ecological perspective, the fact that this profile is made up of primarily educated men with an immigrant background may represent a form of double-bind in which some groups may feel alienated because official discourses and stances about equity in Canada are contradicted by daily life experiences. This group’s pattern of digital media use may be related to the hardships, grievances and social deprivation experienced by minorities both online and offline. The combination of negative life experiences with high emotional distress may lead to experience overall negative and conflictual online social and political exchanges, subsequently legitimazing violence as an ultimate solution [16, 17, 63]. Besides reporting low income similarly to the *Online relational and political engagement/Social media trust* group, the *Limited Internet use/Low trust* profile included less educated and more unemployed participants compared to all other profiles, mostly without an immigration background. Participants in this group may not be content with their socio-political reality, and disengage from social and political issues, at least online. Noteworthy, our profiles suggest that digital media use is closely intertwined with social experiences offline. Interventions should consider this complex interaction and adopt a socio-ecological approach to both research and intervention, tailored not only to the different groups in society but also addressing the gap between them to mend the social fabric.

With regards to depressive symptoms and support for VR, the *Online relational and political engagement/Social media trust* reported the highest levels of depression and support for VR, followed by the *Limited Internet use/Low trust* profile. The fact that the two groups that reported less trust in institutional sources of information compared to alternative social media showed more depressive symptoms and support for VR indicates that issues of trust are important to address with young people in prevention and intervention efforts. Given that individuals in these groups had overall a lower status in society, compared to the other two profiles, it is possible that they may have been experiencing more social deprivation and grievances during the pandemic and have been more sensitive to the anti-system rhetoric which provided meaning to this perceived injustice [60]. This divide aligns with the emergence of polarized social movements in the whole of Canada (e.g., pro- and anti-vaxx groups during the pandemic). Promoting a sense of agency and belonging as well as ensuring that young people can express their opinions and have a purpose in life may help decrease depressive symptoms and reduce overall socio-political distrust and disengement both online and offline, which can in turn contribute to reduce the legitimation of violence. However, such interventions need to consider the social adversity and deprivation experienced by young people and be tailored to the specific needs and challenges that they face. Multi-level systemic interventions that target online and offline socio-political macro-determinants of mental health and injustices in our societies are needed above and beyond individual intervention programs.

The association between membership to the *Online relational and political engagement/Social media trust* profile and support for VR aligns with prior studies pointing to an association between active online political engagement and interactions and support for VR [8, 23, 35]. Noteworthy, this was a characteristic that clearly distinguished the *Online active political engagement/Social media trust* profile from all other profiles. Online relational and political engagement should be addressed in prevention and intervention, while also addressing possible isolation and injustices experienced offline. The association between membership to the *Limited Internet use/Low trust* and support for VR can be related to an overall distrust in society and especially in government and official institutions, which has been found to represent a risk factor for VR [32].

As expected, the group that was at higher risk of supporting VR was also the one that reported the highest level of depressive symptoms, which were significantly and positively associated with support for VR, confirming prior evidence [38, 64–67]. Depressive symptoms do not necessarily lead to greater risk of VR [68]. Yet, multiple studies indicate a positive association between depressive symptoms and support for VR [38, 64–67]. Although directionality of associations remain to be established, available evidence suggests that youth who interact more with strangers online [17, 40], who prefer online social interactions [69–71] and who experience more social adversity [14, 67] are at higher risk of depression, which can partly explain the higher scores of depressive symptoms found among the *Online relational and political engagement/Social media trust* profiles*.* Identifying as a man and being younger were also risk factors for support for VR, in line with prior studies [7, 15], underlining the pertinence for future studies to focus on young people and to consider specificities by gender in VR studies [14, 29, 32, 45].

## Limitations

This study has several limitations. Most importantly, the cross-sectional design prevents us from drawing any conclusions about causality. Longitudinal studies are needed to shed light on the trajectories of associations between patterns of digital media use, depressive symptoms and support for VR. Second, our study is based on a convenience sample with a relatively high socio-economic level and education. This means that our results may not be generalizable to a larger, general population of young adults. Nonetheless, our online method of recruitment is appropriate given the sensitivity of the topic and the challenges of conducting research during a pandemic. Third, all data are based on young people’s self-reports and social desirability biases cannot be excluded. Fourth, our measures of digital media use were limited and not comprehensive of the broad range of possible online experiences. Given the rapidly evolving and dynamic aspects of the Internet, the availability of validated measures for different facets of Internet use remains a challenge for future studies. Last, our data were collected during the COVID-19 pandemic in three Canadian provinces, and results cannot be easily generalized to other provinces or countries, nor to a non-pandemic context.

## Conclusion

Despite these limitations, our findings suggest that digital media use, psychological distress and their interaction play a role in processes of VR among young people and need to be situated and understood within a socio-ecological and social justice perspective. Specifically, trust in different sources of information and social and political experiences online are as relevant as the emotional and relational experiences of young people. The dynamic associations among these key elements have to be considered simultanously when reflecting on VR prevention and digital media use among young people. Prevention efforts should be adapted to the needs of specific populations and consider the diversity of their online/offline experiences. Indeed, our results suggests that online experiences are intertwined with offline experiences in society, in particular with grievances, and that an attention to the rapidly evolving socio-political scenario is warranted when designing intervention programs to prevent processes of VR among young people targeting their digital media use. The fact that self-reported news literacy did not differ across profiles questions the pertinence of VR prevention programs that target mainly news literacy skills among youth. Our findings support preliminary results that showed that media literacy did not protect youth from exposure to extremist content online [35] or risks of VR [25]. It has been argued that programs aimed to foster digital literacy may be associated with improved technical competence but leave participants “critically naïve” [72], failing to situate digital competence within the broader socio-political context. Although digital literacies may still be relevant skills to promote among young people, our findings suggest that, when it comes to the prevention of VR processes, critical thinking skills, supportive environments and a social justice approach to intervention may be equally important.

## Data Availability

The datasets generated during and/or analyzed during the current study are available from the corresponding author upon request.
